# An experimental hut study evaluating the impact of pyrethroid-only and PBO nets alone and in combination with pirimiphos-methyl-based IRS in Ethiopia

**DOI:** 10.1186/s12936-022-04263-x

**Published:** 2022-08-20

**Authors:** Delenasaw Yewhalaw, Meshesha Balkew, Endalew Zemene, Sheleme Chibsa, Peter Mumba, Cecilia Flatley, Aklilu Seyoum, Melissa Yoshimizu, Sarah Zohdy, Dereje Dengela, Seth Irish

**Affiliations:** 1 School of Medical Laboratory Sciences, Faculty of Health Sciences, Jimma University, Jimma, Ethiopia; 2 Tropical and Infectious Diseases Research Center, Jimma University, Jimma, Ethiopia; 3 Abt Associates, PMI VectorLink Project Ethiopia, Addis Ababa, Ethiopia; 4 US President’s Malaria Initiative (PMI), Addis Ababa, Ethiopia; 5 Abt Associates, PMI VectorLink Project, Rockville, MD USA; 6 US President’s Malaria Initiative, USAID, Washington, DC USA; 7 US President’s Malaria Initiative, Center for Disease Control and Prevention, Atlanta, GA USA

## Abstract

**Background:**

Pyrethroid resistance observed in populations of malaria vectors is widespread in Ethiopia and could potentially compromise the effectiveness of insecticide-based malaria vector control interventions. In this study, the impact of combining indoor residual spraying (IRS) and insecticide-treated nets (ITNs) on mosquito behaviour and mortality was evaluated using experimental huts.

**Methods:**

A Latin Square Design was employed using six experimental huts to collect entomological data. Human volunteers slept in huts with different types of nets (pyrethroid-only net, PBO net, and untreated net) either with or without IRS (Actellic 300CS). The hut with no IRS and an untreated net served as a negative control. The study was conducted for a total of 54 nights. Both alive and dead mosquitoes were collected from inside nets, in the central rooms and verandah the following morning. Data were analysed using Stata/SE 14.0 software package (College Station, TX, USA).

**Results:**

The personal protection rate of huts with PermaNet® 2.0 alone and PermaNet® 3.0 alone was 33.3% and 50%, respectively. The mean killing effect of huts with PermaNet® 2.0 alone and PermaNet® 3.0 alone was 2% and 49%, respectively. Huts with PermaNet® 2.0 alone and PermaNet® 3.0 alone demonstrated significantly higher excito-repellency than the control hut. However, mosquito mortality in the hut with IRS + untreated net, hut with IRS + PermaNet® 2.0 and hut with IRS + PermaNet® 3.0 were not significantly different from each other (p > 0.05). Additionally, pre-exposure of both the susceptible *Anopheles arabiensis* laboratory strain and wild *Anopheles gambiae* sensu lato to PBO in the cone bioassay tests of Actellic 300CS sprayed surfaces did not reduce mosquito mortality when compared to mortality without pre-exposure to PBO.

**Conclusion:**

Mosquito mortality rates from the huts with IRS alone were similar to mosquito mortality rates from the huts with the combination of vector control intervention tools (IRS + ITNs) and mosquito mortality rates from huts with PBO nets alone were significantly higher than huts with pyrethroid-only nets. The findings of this study help inform studies to be conducted under field condition for decision-making for future selection of cost-effective vector control intervention tools.

**Graphical Abstract:**

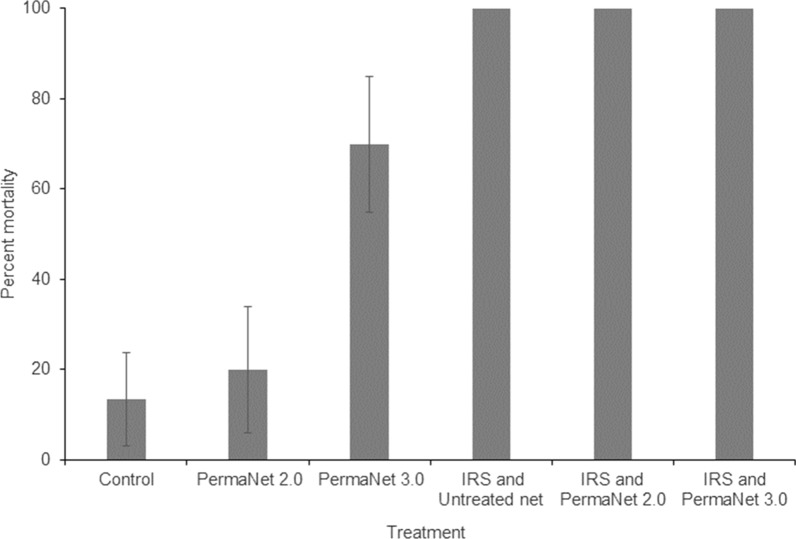

## Background

Malaria is an important public health and socio-economic problem in Ethiopia. The main malaria vector in Ethiopia is *Anopheles arabiensis*, while *Anopheles pharoensis*, *Anopheles funestus* and *Anopheles nili* play a secondary role in malaria transmission. *Anopheles stephensi*, a common and efficient urban malaria vector in Southeast Asia and the Mediterranean Region, has also recently been reported in Ethiopia and neighboring countries including Djibouti and Sudan [1]. There is widespread resistance to dichloro-diphenyl-trichloroethane (DDT) and pyrethroids in *An. arabiensis* and resistance has also been detected to organophosphates in some populations. *Anopheles pharoensis* was also reported to be highly resistant to DDT [2]. The *kdr* mutation (L1014F) has been detected in *An. arabiensis* from many sites, while *ace-1*^*R*^ and *kdr* (L1014S) were not detected in all populations [3–5]. Bottle bioassay tests with *An. arabiensis* populations from all surveyed areas of Ethiopia found resistance to both permethrin and deltamethrin. However, susceptibility to permethrin and deltamethrin was restored following pre-exposure of mosquitoes to the synergist piperonyl butoxide (PBO), indicating involvement of P450-based metabolic resistance mechanisms in the Ethiopian populations of *An. arabiensis* [6].

Indoor residual spraying (IRS) and insecticide-treated nets (ITNs) are the main vector control interventions in Ethiopia. Ethiopia has a long history of IRS. It began spraying DDT in the late 1950s during the Malaria Eradication Programme and malathion was also used for IRS in a few areas until 2010. Deltamethrin, a pyrethroid insecticide, was used for IRS from 2009 to 2013, and since then, bendiocarb and propoxur (carbamate insecticides) and pirimiphos-methyl (organophosphate insecticide) have been used for IRS in Ethiopia. The PMI VectorLink Ethiopia Project piloted the clothianidin (neonicotinoid)-based insecticides, SumiShield® 50WG and Fludora Fusion WP-SB, in 2020 in Menge District in the Benishangul Gumuz Region. Insecticide-treated nets have been widely distributed to households in malaria risk areas every 3 years mainly through the health extension programme [7].

Recent studies in Tanzania showed that the impact of PBO nets could be comparable to the impact of pyrethroid-only nets when combined with IRS, and that there was no significant difference in malaria prevalence between the pyrethroid-only net + IRS and PBO net + IRS study arms [8]. This is of interest for malaria control operations in Ethiopia, as such data can inform future decisions on ITN procurements and strategic distribution, as well as insecticide choices for IRS, which may reduce overall malaria vector control costs. In addition, it is also important to understand possible antagonistic effects of PBO and pirimiphos-methyl, as suggested by the World Health Organization (WHO), before recommending the combined use of PBO nets and pirimiphos methyl IRS for malaria control in Ethiopia.

This study aimed at assessing the impact of the combination of currently available vector control tools (IRS, pyrethroid-only nets, and PBO nets) on mosquito behaviour, mortality and the possible antagonistic effect of PBO and pirimiphos-methyl using experimental huts in Asendabo, Ethiopia. The hypothesis for the Ethiopia context was that the use of PBO nets in combination with IRS would better control malaria vectors than PBO nets alone and that the use of IRS in combination with pyrethroid-only nets would better control malaria vectors than pyrethroid-only nets alone.

## Methods

### Study area

The study was conducted using six experimental huts located at Asendabo, Jimma Zone, Oromia Regional State, Ethiopia, located between latitudes 7° 42′ 50″ N and 07° 53′ 50″ N and longitudes 37° 11′ 22″ E and 37° 20′ 36″ E, at altitudes ranging from 1672 to 1864 m above sea level. The huts were used previously for evaluation of vector control tools [9], but the roofs, walls, floors, ceilings and verandahs of each hut were completely renovated for use in this study. The huts are located approximately 0.3 km from the reservoir shore of Gilgel Gibe hydroelectric power dam in Nada District, Oromia Regional State, Ethiopia. Earlier work showed that *An. arabiensis* population from the study area was resistant to DDT, pyrethroids and malathion, while they were susceptible to primiphos-methyl and some of the carbamates used in malaria vector control [10].

The six treatments in this study included:

1. Control hut (no IRS, untreated net only).
2. Hut with PermaNet® 2.0 (deltamethrin) only (no IRS).
3. Hut with PermaNet® 3.0 (deltamethrin with PBO) only (no IRS).
4. Hut sprayed with Actellic 300CS (1 g/m^2^) and untreated net.
5. Hut sprayed with Actellic 300CS (1 g/m^2^) and PermaNet® 2.0.
6. Hut sprayed with Actellic 300CS (1 g/m^2^) and PermaNet® 3.0.

### Experimental huts

The huts used for this trial were the West African type [11]. They were built approximately 3 m apart from one another on a concrete foundation which has a moat filled with water to prevent ants and other scavengers that may potentially enter the hut and feed on dead mosquitoes. The walls were made of bricks and the roofs of corrugated iron. Each hut had four louvred windows made of iron sheets with 1 cm between slits which allows free flying mosquitoes to enter the hut but restricts them from exiting the hut. A ceiling made of white sheet cloth allowed for easy capture of mosquitoes resting inside the huts. Huts had a central room and a verandah. The verandah was left open from the inside to allow for entry of mosquitoes from the central room and the walls were screened partly with white plastic sheets and partly with metal mesh to capture exiting mosquitoes.

### Use of nets for the study

During the study, two types of nets (PermaNet® 2.0 and PermaNet® 3.0) with and without IRS were assessed. Moreover, an untreated net was used with IRS and untreated net without IRS was used as a negative control. Both PermaNet® 2.0 and PermaNet® 3.0 nets are treated with the pyrethroid deltamethrin, but PermaNet 3.0 has also PBO synergist. During the study, each ITN in each hut was replaced by a new replicate net every 3 weeks since the trial had no replicate huts. Eighteen holes (4 cm × 4 cm) were cut in each net purposefully to simulate a torn net [11].

### Indoor residual spraying (IRS)

Actellic® 300CS, a pirimiphos-methyl-based IRS insecticide, was applied to the three IRS treatment huts 1 week before the start of the trial. Pirimiphos-methyl is an organophosphate insecticide being used for IRS by the malaria elimination program of Ethiopia. During the spraying, filter papers were affixed to the wall surfaces at three heights (high, middle and low) to ensure the application was properly and uniformly applied and determine the concentration of the applied insecticide. The spray operators were trained on the spraying techniques and the wall surfaces of huts were lined with chalk to properly maintain the swath and avoid overlap following the WHO protocol.

### Mosquito collection

Mosquitoes were collected 6 days per week over 9 weeks (from 16th November 2020 to 16th January 2021), for a total of 54 collection nights from each of the six experimental huts following the WHO Pesticide Evaluation Scheme (WHOPES) recommendations [11].

Six volunteer mosquito collectors from the local community were recruited and trained to sleep in each hut every night for a total of 54 nights provided with a bed and mattress every night. Sleepers were rotated among the six huts every night, completing one rotation every week. This was done to address differential mosquito attraction to individual sleepers. The sleepers entered the huts each evening at 6:00 p.m. and started mosquito collection in each hut at dawn at 6:00 a.m.

At 6:00 a.m., mosquito collection (both dead and alive) commenced using mouth aspiration, starting from inside the nets, then from inside the hut (floor, wall, and ceilings) and finally from the verandah. Mosquitoes were sorted, counted, and scored by location (inside net, inside the central room of the hut, and verandah), physiological stage (unfed or blood-fed) and whether they were dead or alive. Mosquitoes were identified using the standard African *Anopheles* morphological key [12]. Dead mosquitoes were kept in Eppendorf tubes over silica gel until further processing in the laboratory. Live mosquitoes were kept in paper cups in an insecticide-free room and provided with 10% sugar solution for 24 h at which point delayed mortality was recorded.

### Wall bioassay

Cone bioassays were conducted twice during the trial period to assess the bio-efficacy of Actellic 300CS. At each time point, three cones were fixed to the wall surfaces at three heights from the ground (high 160 cm, medium 100 cm and low 40 cm) of each hut and ten mosquitoes were used per cone with a total of 30 mosquitoes per hut, for each of the three sprayed huts and a control hut (120 mosquitoes in total). Mosquitoes were exposed for 30 min after which immediate knockdown was recorded and mortality was recorded after a 24 h holding period. Non-blood-fed, 3–5 day old adult female *Anopheles gambiae* sensu lato (s.l.) were used for bioassays.

The first cone bioassay was conducted 3 weeks after spray using a confirmed susceptible *An. arabiensis* strain from Sekoru Insectary without pre-exposure to PBO synergist. The second bioassay was conducted at the end of the trial (10 weeks after spray). In the second bioassay, both susceptible *An. arabiensis* laboratory strain from Sekoru Insectary and wild *An. gambiae* s.l. were used. The assay was carried out with and without PBO pre-exposure for both strains. Wild populations of *An. gambiae* s.l. used for the bioassays were raised from larvae and pupae collected from semi-permanent and permanent breeding habitats around Wolkite area, central Ethiopia. They were reared to adults at Asendabo Field Insectary. Mosquitoes were exposed to papers treated with PBO (2%) in a WHO cylinder for 1 h.

### Blood meal host source analysis and mosquito identification

All the fed mosquitoes collected from the different huts were assayed to determine the blood meal host source using direct ELISA [13]. Moreover, a sub-sample of *An. gambiae* s.l. was also molecularly identified to species using species-specific PCR following established protocol [14].

### Variables

Four study outcomes measured were:

i. Deterrence—the reduction in hut entry by mosquitoes in treatment huts relative to the control hut (hut with untreated net and no IRS).
ii. Exophily—the proportion of mosquitoes found resting on the walls of the verandah.
iii. Blood-feeding inhibition—the reduction in blood-feeding in treatment huts in comparison with control hut (hut with untreated net and no IRS).
iv. Immediate and delayed mortality—the proportion of mosquitoes entering the hut that were dead in the morning (immediate mortality) or after being caught alive and held for 24 h with access to a sugar solution (delayed mortality).

### Data analysis

Data from the five treatment huts were compared with data from the control hut to determine the insecticidal effects on the variables listed above.

Personal protection, killing effect, and exophily were estimated using the following formulas [9, 11].

$${\text{Personal}}\;{\text{protection}}\;(\%)=100\times(B_u-B_t)/B_u;$$

where *B*_*t*_ is the total number of freshly blood-fed mosquitoes in the huts with treated nets. *B*_*u*_ is the total number of freshly blood-fed mosquitoes in the huts with untreated nets.

$$\text{Killing}\;{\text{effect}}\;(\%)=100\times(K_t-K_u)/T_u;$$

where *K*_*t*_ is number of mosquitoes killed (immediate) in the huts with treated nets. *K*_*u*_ is number of mosquitoes killed (immediate) in the huts with untreated nets. *T*_*u*_ is the total number of mosquitoes collected from the huts with untreated nets.

$${\text{Exophily}}\;(\%)=({\text{E}}_{\text{v}}/{\text{E}}_{\text{t}})\times100;$$

where E_v_ is the number of mosquitoes found in verandah. E_t_ is the total number of mosquitoes found inside the hut and verandah

For all three parameters, mosquito entry was estimated by adding the number of dead and alive mosquitoes observed inside the hut and the verandah. Estimates of personal protection and killing effect were determined using mosquitoes from all collection nights.

To test differences in mean mosquito density in each of the treatment huts, ANOVA was employed after log-transformation of non-normal distributed mosquito count data. T-tests were employed to determine the differences in mean proportions of blood-fed mosquitoes and mosquitoes exiting early from the different test huts and control hut.

In all analyses, the hut with the untreated net and without IRS was used as a negative control, but comparisons were made between all treatment groups. In all analyses, individual huts and sleepers were included in the models as random effects.

## Results

**Fig. 1:**
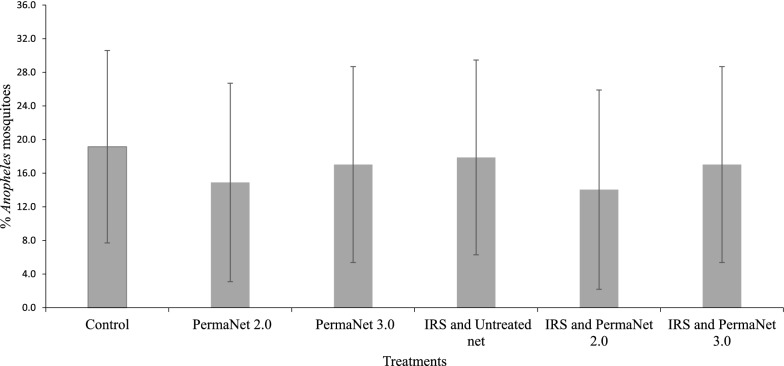
Proportion of mosquitoes collected that were *Anopheles* spp. in each experimental hut, Asendabo, Ethiopia. Control is no IRS and untreated net

Overall, a total of 386 mosquitoes, comprising 235 *Anopheles* spp. (60.9%) and 151 *Culex* spp. (39.1%), were collected during the study. Of the *Anopheles* mosquitoes, *An. gambiae* s.l., *An. pharoensis* and *Anopheles coustani* accounted for 228 (97.0%), 6 (2.6%) and 1 (0.4%), respectively. The proportion of the total *Anopheles* mosquitoes collected from each hut is presented in Fig. 1. There were no significant differences in mean percentage of *Anopheles* mosquitoes between the different treatment and control huts (p > 0.05).

**Fig. 2:**
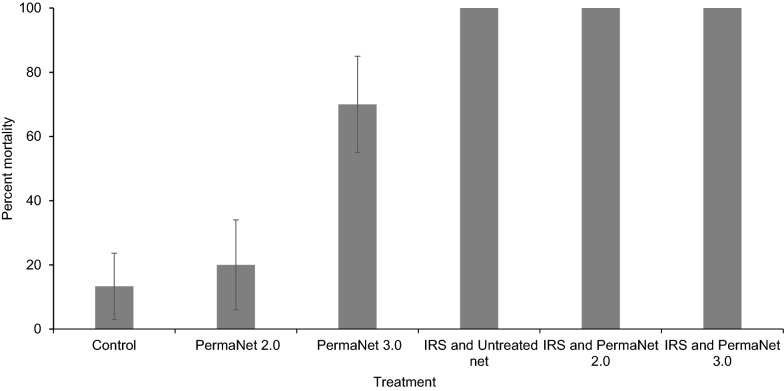
Proportion of immediate mortality of *Anopheles* mosquitoes collected from each experimental hut, asendabo, Ethiopia

An immediate mosquito mortality of 100% was recorded from all three huts with IRS throughout the study period. The mean immediate mosquito mortality rates from the control, PermaNet® 2.0 and PermaNet® 3.0 huts were 13.3% (95% CI 3–23%), 20.0% (95% CI 6–33%) and 70.0% (95% CI 55–84%), respectively (Fig. 2). The immediate mosquito mortality rate from the hut with IRS + untreated net was significantly higher than the PermaNet® 3.0 hut (p < 0.001). Similarly, the immediate mosquito mortality rate from the hut with PermaNet® 3.0 was significantly higher than that from the control hut (p < 0.001) as well as the PermaNet® 2.0 (p < 0.001) hut. The mosquito mortality rate from the PermaNet® 2.0 hut was not significantly different from that of the control hut (p = 0.4). On average the killing effect of PermaNet® 2.0 and PermaNet® 3.0 compared to the untreated net were 2.2% and 48.9%, respectively. The killing effects in the huts with PermaNet® 2.0 and PermaNet® 3.0 increased to 60.0% and 75.5% when combined with IRS. Delayed mosquito mortality rates of 14.3% and 41.7% were recorded from huts with PermaNet® 2.0 and PermaNet® 3.0, respectively.

**Fig. 3:**
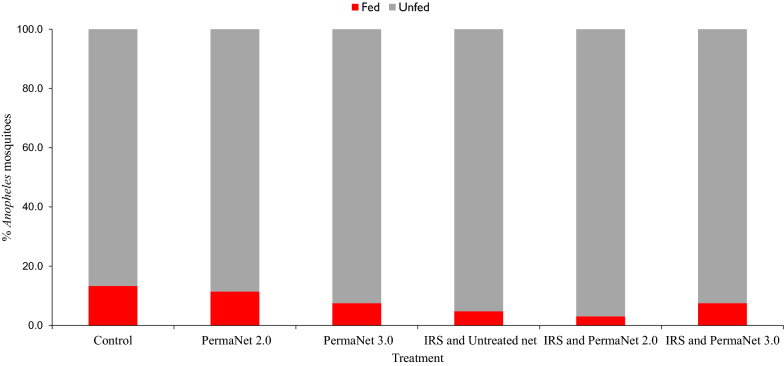
Proportion of fed and unfed *Anopheles* in different experimental huts in Asendabo, Ethiopia

Of the 50 sub-samples of *An. gambiae* s.l. analysed using species-specific PCR, 46 (92%) were identified as *An. arabiensis* and four mosquito samples failed to amplify. The blood-feeding rate of the *Anopheles* collected from each of the huts is presented in Fig. 3. Overall, 26 of the *Anopheles* (11.1%) were blood-fed upon collection. Of the fed mosquitoes, human, bovine and mixed human/bovine blood were detected in 14 (53.9%), 2 (7.7%), 5 (19.2%) of the specimens, respectively. Blood meals from the remaining 5 (19.2%) of the specimens were unidentified. The overall proportion of human blood-fed *Anopheles* was 73.1% (n = 19). The personal protection rates of PermaNet® 2.0 and PermaNet® 3.0 nets were 33.3% and 50%, respectively. When combined with IRS, the personal protection rate of PermaNet® 2.0 increased to 83.3% while the personal protection rate of PermaNet® 3.0 remained the same (50%). The proportions of human blood-fed *Anopheles* collected from the control, PermaNet® 2.0, PermaNet® 3.0 and combined IRS-treated huts were 13.3% (95% CI 3.4–23.3), 11.4% (95% CI 0.9–22.0), 7.5% (95% CI 0.0–15.7) and 5.2% (95% CI 1.2–9.3), respectively.

**Fig. 4:**
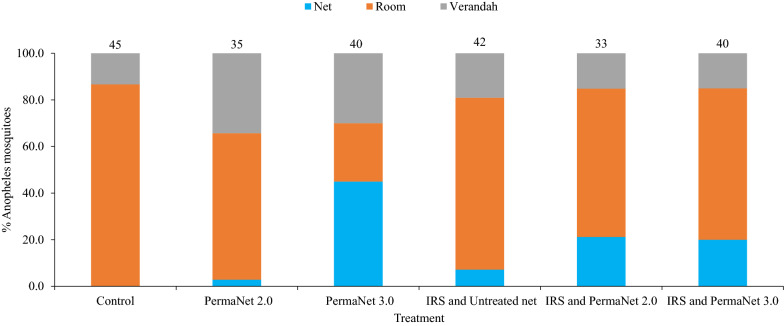
Proportion of *Anopheles* collected from inside nets, rooms and verandahs of different experimental huts, Asendabo, Ethiopia

Overall, the majority of *Anopheles* were collected from the rooms of the huts. The proportions of *Anopheles* collected from the verandah of the IRS-treated and the control huts were 16.5% and 13.3%, respectively (Fig. 4). The proportion of *Anopheles* collected from verandah of a hut with PermaNet® 2.0 was significantly higher than the proportion of *Anopheles* collected from the control hut (p = 0.02).

**Fig. 5:**
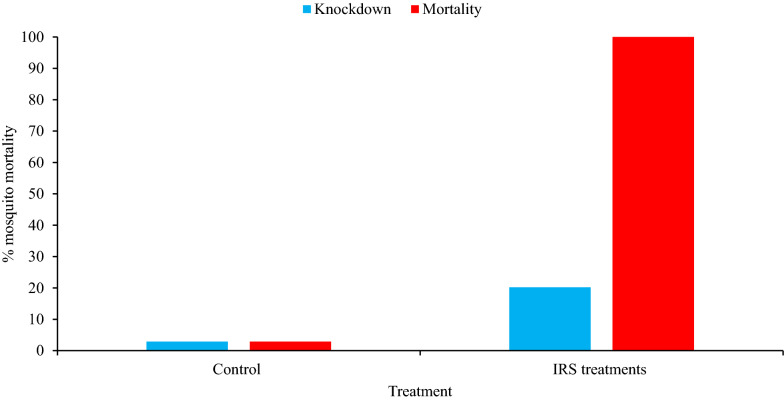
Mean 30 min knockdown and 24 h mortality rates of the susceptible *An. arabiensis* laboratory strain following wall bioassays 3 weeks after Actellic 300CS spraying, Asendabo, Ethiopia

The mean knockdown rates of the susceptible *An. arabiensis* laboratory strain in the bioassays conducted 3 weeks post-spray were 20.2% and 2.9% in the IRS-treated and control huts, respectively. The mean 24 h mortality rates from IRS-treated and control huts were 100% and 2.9%, respectively (Fig. 5).

**Fig. 6:**
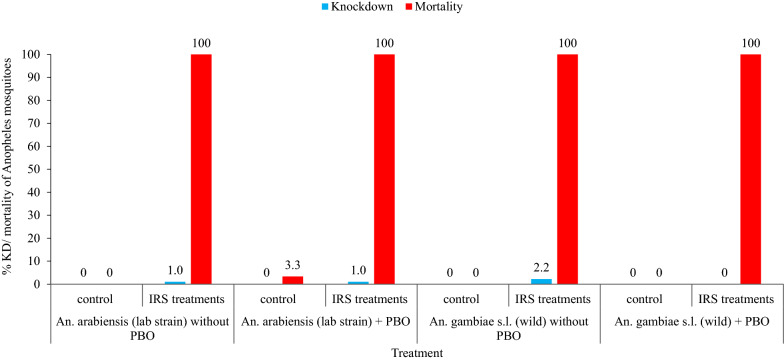
Mean 30 min knockdown and 24 h mortality rates of the susceptible *An. arabiensis* laboratory strain and wild *An. gambiae* s.l. (with and without pre-exposure to PBO) following wall bioassays 10 weeks after IRS using Actellic 300CS, Asendabo, Ethiopia

The combined mean 30 min knockdown rate of the *An. arabiensis* laboratory strain and wild population of *An. gambiae* s.l. exposed to IRS-treated huts 10 weeks post-spray, with and without pre-exposure to PBO, was 1.1% (Fig. 6). The 24 h mortality rate of the *An. arabiensis* laboratory strain and wild population of *An. gambiae* s.l. at 10 weeks post-spray which were exposed to IRS-treated hut wall surfaces were 100%, irrespective of pre-exposure of mosquitoes to PBO.

## Discussion

Malaria vector control interventions have been intensified over the last decade in Ethiopia resulting in remarkable progress, with a reduction in children under-five mortality rates from 123 deaths per 1000 live births in 2005 to 55 deaths per 1000 live births in 2019 [15]. The vector control activities including mass-distribution/replacement of ITNs every 3 years in accordance with WHO recommendations and deployment of IRS in targeted areas appear to have played vital roles in the successes obtained in malaria control in Ethiopia [16]. However, malaria still remains a public health challenge in the country, where an estimated 52% of the population lives in areas at risk of malaria. With the successes gained in malaria control, a national malaria elimination goal is set for 2030 [17]. This study evaluated the impact of combining currently available vector control tools on mosquito behaviour and mortality.

Compared to PermaNet® 2.0, PermaNet® 3.0 is impregnated with a higher concentration of deltamethrin and the roof panel is treated with both deltamethrin and PBO. The higher killing effect of PermaNet® 3.0 compared to PermaNet® 2.0 in this study could be attributed to the presence of the PBO synergist on the roof panel of the net and/or higher dosage of deltamethrin in the net. The synergist PBO works to inhibit P450 detoxification of pyrethroid insecticides and can result in partial or complete restoration of susceptibility to certain pyrethroids, such as deltamethrin, hence enhancing efficacy of the insecticides [18]. The role of PBO in affecting susceptibility in both wild and laboratory strain mosquitoes exposed to Actellic® 300CS sprayed walls could not be determined as mortality rates in all IRS treatment huts were 100%. PBO nets have been shown to provide high levels of protection against *An. gambiae* in a recent experimental hut trial in Côte d’Ivoire [19]. Moreover, the improved protective role of PBO nets on malaria prevalence was also documented in Uganda [20]. Personal protective efficacy of PBO nets may also last longer compared to the standard nets [21]. PBO nets are particularly important as a malaria control tool in areas where pyrethroid resistance is high in the vector populations [22] and is, therefore, under consideration for more widespread distribution in Ethiopia. Despite these added advantages of PBO nets, the cost is currently higher than pyrethroid nets which may limit their wider use in malaria vector control in malaria endemic areas. Additionally, this study provides an example of a setting with high levels of pyrethroid resistance where PBO nets may not be sufficient.

The proportion of *Anopheles* retrieved from the verandah of huts with both PermaNet 2.0 and PermaNet 3.0 was higher than those from the verandah of the control hut. Huts with PermaNet 2.0 induced significantly higher exito-repellency compared to control huts. Insecticides often induce toxicity to mosquitoes. However, the non-toxic ‘exito-repellent’ effects of insecticides are often less understood. Pyrethroid and organophosphate insecticides are also known to have these non-killing effects. The impact of the non-killing effects of the insecticides on the risk of malaria is not clearly understood. Though exito-repellency appears to reduce indoor human-vector contact, it may on the contrary enhance risk of outdoor mosquito biting [23].

The role of IRS as a key malaria vector control intervention tool is evident. Immediate mortality was observed in mosquitoes collected from the three huts sprayed with Actellic® 300CS up to the end of the study at 10 weeks post-spray. The proportion of blood-fed *Anopheles* was significantly lower in the IRS-treated huts compared to the control hut. This could be due to the fast-acting nature of Actellic® 300CS insecticide with both contact and fumigant effects. This shows the potential of Actellic® 300CS as an insecticide of choice for IRS in areas where the local vector population is susceptible to the insecticide. *Anopheles arabiensis* is susceptible to pirimiphos-methyl in most parts of Ethiopia [10, 24]. Some earlier studies conducted in Ethiopia showed the effectiveness of combining IRS with ITNs compared to ITNs alone in suppressing vector populations [25]. Added benefits of combining IRS and ITNs in malaria prevention was also reported from other areas in sub-Saharan Africa [26–28]. In contrast, limited or no added benefit of combining IRS with ITNs as compared to ITNs alone has been reported elsewhere [29, 30]. This inconsistency could be attributed to differences in the intensity of malaria transmission and insecticide resistance of the local vector populations. The impact of the combined use of available vector control interventions appears to be more pronounced when applied in high transmission areas, and where the local vector populations are susceptible to the insecticide used for the IRS component [25].

The importance of combining IRS and ITNs for malaria vector control is believed partly to come from the low or inconsistent utilization of ITNs by users and short residual efficacy of IRS insecticides [31, 32]. In Ethiopia, household surveys have shown approximately 50% of children under five and pregnant women use their nets with geographic variation [33]. The gap often created as a result of low utilization of ITNs may be complemented with IRS. ITN utilization and care depends on personal decisions and behavior [34]. However, once properly implemented, IRS rapidly suppresses local vector populations with no additional behaviors required from the residents, hence reducing malaria incidence [35]. However, effectiveness of IRS for malaria vector control is highly affected by both physiological and behavioral resistance of the local vector population to the insecticide [36], apart from operational factors and higher costs.

As the study was conducted during the dry season of the year, the mosquito population density was not high and, therefore, limits the generalizability of the results. However, the findings of this study could provide preliminary information for vector control programs for the rational choice of future vector control intervention tools. In addition, combining indoor residual spraying using pirimiphos-methyl with PBO nets has been reported to be antagonistic in some studies, but a negative interaction or antagonism between PBO and pirimiphos-methyl was not detected in this study.

## Conclusion

In this study, significantly higher mosquito mortality rates were recorded from treatment huts with IRS than huts with ITNs alone, regardless of net type. Interestingly, the hut with IRS + untreated net had similar mosquito mortality rates as huts with combinations of vector control tools (IRS + pyrethroid-only nets and IRS + PBO nets). Moreover, immediate mortality ratre of mosquitoes in IRS + untreated net was significantly higher than PermaNet 2.0 and PermaNet 3.0 nets. These suggest that IRS alone provides the greatest vector control impact. Indoor residual spraying alone or in combination with ITNs offered significantly higher personal protection against mosquito bites than the control hut. Moreover, the proportion of mosquito mortality was significantly higher in huts with PBO nets than huts with pyrethroid-only nets, but remained significantly lower than the huts that received IRS. In general, the findings of this study revealed that IRS alone was as efficacious as combinations of vector tools (IRS + ITNs) for malaria vector control at least over the 9-week study period. Field studies to further assess the impact of combinations of vector tools on malaria vector control in different eco-epidemiological settings are also warranted. This study provides additional data for considering future selection of the most appropriate vector control tools for improved malaria vector control and management of insecticide resistance. This study also indicated that there was no antagonistic effect between PBO and IRS with pirimiphos-methyl. However, further studies may be needed to determine if there are antagonistic effects in large scale IRS applications to evaluate the efficacy of PBO vs. IRS against different resistant vector populations in Ethiopia for decision-making to deploy either PBO nets or pirimiphos-based IRS intervention.

## Data Availability

Data supporting the conclusions of this article are included within the article.

## Abbreviations

DDT: Dichloro-diphenyl-trichloroethane
ELISA: Enzyme-linked Immunosorbent Assay
IRS: Indoor residual spray
ITN: Insecticide-treated net
PBO: Piperonyl butoxide
PCR: Polymerase chain reaction
WHO: World Health Organization
WHOPES: World Health Organization Pesticide Evaluation Scheme

## References

[1] Sinka ME, Pirononb S, Masseyc NC, Longbottomd J, Hemingway J, Moyesc JCL, Willis KJ (2020). A new malaria vector in Africa: predicting the expansion range of *Anopheles stephensi* and identifying the urban populations at risk. Proc Natl Acad Sci USA.

[2] Balkew M, Elhassen I, Ibrahim M, Gebre-Michael T, Engers H (2006). Very high DDT-resistant population of *Anopheles pharoensis* Theobald (Diptera: Culicidae) from Gorgora, northern Ethiopia. Parasite.

[3] Balkew M, Ibrahim M, Koekemoer LL, Brooke BD, Engers H, Aseffa A (2010). Insecticide resistance in *Anopheles arabiensis* (Diptera: Culicidae) from villages in central, northern and south west Ethiopia and detection of kdr mutation. Parasites Vectors.

[4] Yewhalaw D, Van Bortel W, Denis L, Coosemans M, Duchateau L, Speybroeck N (2010). First evidence of high knockdown resistance frequency in *Anopheles arabiensis* (Diptera: Culicidae) from Ethiopia. Am J Trop Med Hyg.

[5] Yewhalaw D, Wassie F, Steurbaut W, Spanoghe P, Van Bortel W, Denis L (2011). Multiple insecticide resistance: an impediment to insecticide-based malaria vector control program. PLoS ONE.

[6] Yewhalaw D, Asale A, Tushune K, Getachew Y, Duchateau L, Speybroeck N (2012). Bio-efficacy of selected long-lasting insecticidal nets against pyrethroid resistant *Anopheles arabiensis* from South-Western Ethiopia. Parasites Vectors.

[7] PMI. Malaria operational plan FY 2018—Ethiopia. USAID. 2018.

[8] Protopopoff N, Mosha JF, Lukole E, Charlwood JD, Wright A, Mwalimu CD (2018). Effectiveness of a long-lasting piperonyl butoxide-treated insecticidal net and indoor residual spray interventions, separately and together, against malaria transmitted by pyrethroid-resistant mosquitoes: a cluster, randomised controlled, two-by-two factorial design trial. Lancet.

[9] Asale A, Getachew Y, Hailesilassie W, Speybroeck N, Duchateau L, Yewhalaw D (2014). Evaluation of the efficacy of DDT indoor residual spraying and long-lasting insecticidal nets against insecticide resistant population of *Anopheles arabiensis* Patton (Diptera: Culicidae) from Ethiopia using experimental huts. Parasites Vectors.

[10] Alemayehu E, Asale A, Eba K, Getahun K, Tushune K, Bryon A (2017). Mapping insecticide resistance and characterization of resistance mechanisms in Anopheles arabiensis (Diptera: Culicidae) in Ethiopia. Parasites Vectors.

[11] WHO (2013). Guidelines for laboratory and field-testing of long-lasting insecticidal nets.

[12] Coetzee M (2020). Key to the females of Afrotropical *Anopheles* mosquitoes (Diptera: Culicidae). Malar J.

[13] Beier JC, Perkins PV, Wirtz RA, Koros J, Diggs D, Gargan TP (1988). Bloodmeal identifcation by direct enzyme-linked immunosorbent assay (ELISA), tested on *Anopheles* (Diptera: Culicidae) in Kenya. J Med Entomol.

[14] Scott JA, Brogdon WG, Collins FH (1993). Identifcation of single specimens of the *Anopheles gambiae* complex by the polymerase chain reaction. Am J Trop Med Hyg.

[15] EPHI and ICF. Ethiopia mini demographic and health survey 2019: final report. Rockville: EPHI and ICF.

[16] Bekele D, Belyhun Y, Petros B, Deressa W (2012). Assessment of the effect of insecticide-treated nets and indoor residual spraying for malaria control in three rural kebeles of Adami Tulu District, South Central Ethiopia. Malar J.

[17] FMOH (2018). National malaria guidelines.

[18] Ketoh GK, Ahadji-Dabla KM, Chabi J, Amoudji AD, Apetogbo GY, Awokou F (2018). Efficacy of two PBO long-lasting insecticidal nets against natural populations of *Anopheles gambiae* s. l. in experimental huts, Kolokopé, Togo. PLoS ONE.

[19] Oumbouke WA, Rowland M, Koffi AA, Alou LP, Camara S, N’Guessan R (2019). Evaluation of an alpha-cypermethrin + PBO mixture long-lasting insecticidal net VEERALIN® LN against pyrethroid resistant *Anopheles gambiae* s.s.: an experimental hut trial in M’bé, central Côte d’Ivoire. Parasites Vectors.

[20] Staedke SG, Gonahasa S, Dorsey G, Kamya MR, Maiteki-Sebuguzi C, Lynd A (2020). Effect of long-lasting insecticidal nets with and without piperonyl butoxide on malaria indicators in Uganda (LLINEUP): a pragmatic, cluster-randomised trial embedded in a national LLIN distribution campaign. Lancet.

[21] Martin JL, Mosha FW, Lukole E, Rowland M, Todd J, Charlwood JD (2021). Personal protection with PBO-pyrethroid synergist-treated nets after 2 years of household use against pyrethroid-resistant Anopheles in Tanzania. Parasites Vectors.

[22] Gleave K, Lissenden N, Richardson M, Choi L, Ranson H (2021). Piperonyl butoxide (PBO) combined with pyrethroids in insecticide-treated nets to prevent malaria in Africa. Cochrane Database Syst Rev.

[23] Achee NL, Sardelis MR, Dusfour I, Chauhan KR, Grieco JP (2009). Characterization of spatial repellent, contact irritant, and toxicant chemical actions of standard vector control compounds. J Am Mosq Control Assoc.

[24] Messenger LA, Shililu J, Irish SR, Anshebo GY, Tesfaye AG, Ye-Ebiyo Y (2017). Insecticide resistance in *Anopheles arabiensis* from Ethiopia (2012–2016): a nationwide study for insecticide resistance monitoring. Malar J.

[25] Kenea O, Balkew M, Tekie H, Deressa W, Loha E, Lindtjørn B (2019). Impact of combining indoor residual spraying and long-lasting insecticidal nets on *Anopheles arabiensis* in Ethiopia: results from a cluster randomized controlled trial. Malar J.

[26] West PA, Protopopoff N, Wright A, Kivaju Z, Tigererwa R, Mosha FW (2014). Indoor residual spraying in combination with insecticide-treated nets compared to insecticide-treated nets alone for protection against malaria: a cluster randomised trial in Tanzania. PLoS Med.

[27] Hamel MJ, Otieno P, Bayoh N, Kariuki S, Were V, Marwanga D (2011). The combination of indoor residual spraying and insecticide-treated nets provides added protection against malaria compared with insecticide-treated nets alone. Am J Trop Med Hyg.

[28] Oguttu DW, Matovu JK, Okumu DC, Ario AR, Okullo AE, Opigo J (2017). Rapid reduction of malaria following introduction of vector control interventions in Tororo District, Uganda: a descriptive study. Malar J.

[29] Corbel V, Akogbeto M, Damien GB, Djenontin A, Chandre F, Rogier C (2012). Combination of malaria vector control interventions in pyrethroid resistance area in Benin: a cluster randomised controlled trial. Lancet Infect Dis.

[30] Loha E, Deressa W, Gari T, Balkew M, Kenea O, Solomon T (2019). Long-lasting insecticidal nets and indoor residual spraying may not be sufficient to eliminate malaria in a low malaria incidence area: results from a cluster randomized controlled trial in Ethiopia. Malar J.

[31] Admasie A, Zemba A, Paulos W (2018). Insecticide-treated nets utilization and associated factors among under-5 years old children in Mirab-Abaya District, Gamo-Gofa Zone, Ethiopia. Front Public Health.

[32] Yitayew AE, Enyew HD, Goshu YA (2018). Utilization and associated factors of insecticide treated bed net among pregnant women attending antenatal clinic of Addis Zemen Hospital, North-Western Ethiopia: an institutional based study. Malar Res Treat.

[33] FMoH (2016). Ethiopia national malaria indicator survey 2015.

[34] Baume CA, Reithinger R, Woldehanna S (2009). Factors associated with use and non-use of mosquito nets owned in Oromia and Amhara regional states, Ethiopia. Malar J.

[35] Abong’o B, Gimnig JE, Torr SJ, Longman B, Omoke D, Muchoki M (2020). Impact of indoor residual spraying with pirimiphos-methyl (Actellic 300CS) on entomological indicators of transmission and malaria case burden in Migori County, western Kenya. Sci Rep.

[36] Russell TL, Beebe NW, Cooper RD, Lobo NF, Burkot TR (2013). Successful malaria elimination strategies require interventions that target changing vector behaviours. Malar J.

